# Extended high-frequency audiometry in healthy adults with different age groups

**DOI:** 10.1186/s40463-021-00534-w

**Published:** 2021-08-26

**Authors:** Mingming Wang, Yu Ai, Yuechen Han, Zhaomin Fan, Peng Shi, Haibo Wang

**Affiliations:** 1 Department of Otorhinolaryngology Head and Neck Surgery, Shandong Provincial ENT Hospital, Cheeloo College of Medicine, Shandong University, Jinan, China; 2 Department of Clinical Audiology, Shandong Provincial ENT Hospital, Cheeloo College of Medicine, Shandong University, Jinan, China; 3 Department of Breast and Thyroid Surgery, Shandong Provincial Hospital Affiliated to Shandong First Medical University, 324 Jingwu Road, Jinan, China

**Keywords:** Hearing threshold, Extended high frequency, Age

## Abstract

**Background:**

It was well-documented that extended high-frequency (EHF, above 8 kHz) hearing test could be more sensitive comparing with the conventional measurement on frequency below 8 kHz, regarding the early prediction of auditory damage in certain population. However, hardly any age-specific thresholds of EHF in population with normal hearing ability were observed. This study aims to monitor the age-dependent hearing thresholds at EHF (from 9 to 20 kHz) in healthy hearing population.

**Methods:**

A total of 162 healthy participants (from 21 to 70 years) with normal conventional pure tone audiograms were recruited and separated into five groups by age. Conventional pure tone average was performed with frequencies from 0.25 to 8 kHz under air conduction and from 0.25 to 4 kHz under bone conduction. EHF audiometry from 9 to 20 kHz was determined under air conduction.

**Results:**

The effects of aging on hearing were evident at frequencies above 4 kHz. The hearing thresholds of EHF were less than 26 dB HL before 30 years-olds. Hearing abilities in EHF were deteriorated starting from the 31 ~ 40 group and were most obvious in the 51 ~ 60 group and the 61 ~ 70 group with the maximum thresholds of 75 dB HL. Sensitivity of EHF was inversely proportional to the frequency within each age group, and to age among groups. Subjects under 30 years old were totally responsive up to 16 kHz, and 52.2% could respond to 20 kHz. Meanwhile, no responsiveness was recorded to 20 kHz in the 51 ~ 60 group and even to 18 kHz in the 61 ~ 70 group. No gender differences in hearing threshold was observed within each age group, except an obvious decline at frequencies of 4, 6, 8, and 9 kHz in male participants of the 41 ~ 50 group.

**Conclusions:**

Hearing thresholds at EHF from 9 to 20 kHz were more sensitive than at frequencies below 8 kHz for hearing measurement, and aging inversely affected hearing ability at EHF in healthy population. Hearing thresholds at EHF deteriorated with age and raising frequency, while the upper frequency limit decreased with aging.

**Graphical abstract:**

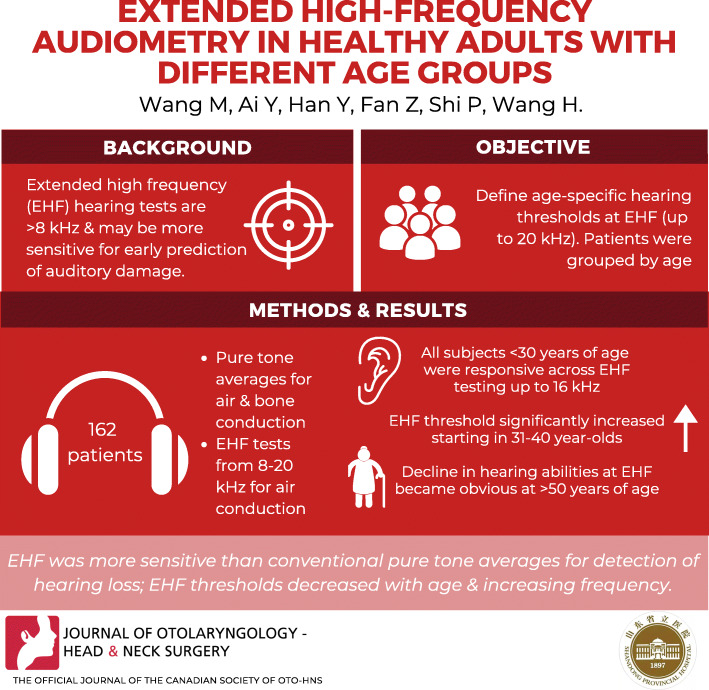

## Introduction

The conventional method of measuring pure tone average (PTA) of hearing thresholds is performed under the frequencies lowing than 8 kHz. However, this conventional audiometry has been suggested not reliable on evaluating auditory damage due to the absence of detecting responses at extended high frequencies (EHF) above 8 kHz. Monitoring EHF responses, on the other hand, could be quite useful in the early prediction of hearing loss in certain populations [1–4]. Research has demonstrated that measuring the hearing sensitivity at EHF could be useful for monitoring the auditory function in patients undertaken ototoxic agents, because the changes in EHF hearing occurred prior to changes of conventional measurements at frequencies below 8 kHz [5, 6]. The validity of the use of EHF is still controversial, although there has been some additional research out that supports use of it in detection of the noise-induced hearing loss (NIHL) [2, 7, 8]. Antonioli suggested that the EHF audiometry might not be an optimal tool to identify NIHL [9]. The hearing thresholds at EHF had also been suggested to be a good parameter in predicting difficulty in understanding speech in noise, in patients whose hearing abilities that are deemed to be normal by conventional PTA evaluation [10].

Studies with multivariate analysis proved that age was the primary factor of changes to hearing thresholds at EHF [11, 12]. Aging was tightly correlated with the prediction of hearing impairment, even after the adjustment of other important risking factors. It has been shown that the age-related hearing change appeared first at EHF rather than at the lower frequencies [13]. The sensitivity of hearing at EHF worsened with increased age at a rate faster than that at frequencies below 8 kHz, and the inter-subject variability of pure-tone thresholds at EHF was higher than that at frequencies below 8 kHz [7, 14–17].

With the limitations of interpretations that can be made by conventional PTA, EHF audiometry had been performed more often as hearing parameters for clinical and laboratory diagnoses change with research. However, hardly any age-dependent normal audiometric thresholds at EHF from 9 to 20 kHz had been reported up to now. Hence, the present study aimed to provide the data of hearing thresholds at frequencies up to 20 kHz of different age groups in the health population to serve the need.

## Methods

### Subjects and grouping

Adults with normal PTA hearing thresholds (≤ 20 dB HL, up to 2 kHz), aged between 21 to 70 years old, were recruited in the study. Subjects were in a normal state of health, free from all signs or symptoms of ear diseases. They performed ear canal cleaning before testing. No history of noise exposure (including military, occupational and recreational noise exposures), smoking, intake of alcohol and ototoxic drugs (including loop diuretics, nonsteroidal anti-inflammatory drugs, antibiotics, chemotherapeutic agents, quinine, and acetaminophen) [18], hypertension, diabetes, or familial hearing loss was reported by the subjects. All participants had healthy tympanic membranes, evaluated by otoscopy, and normal middle-ear function by immittance test (pressure between − 50 and 50 daPa, and immittance between 0.3 and 1.6 cc). Participants were grouped according to their age as the following five categories: 21 ~ 30 years, 31 ~ 40 years, 41 ~ 50 years, 51 ~ 60 years, or 61 ~ 70 years. All test procedures were performed using a non-invasive technique, and were explained to all participants before testing. Informed consent was taken from each participant before data collection.

### Hearing assessment

Audiometric tests were conducted by experienced technicians. Hearing thresholds of each ear at 15 frequencies from 0.25 to 20 kHz were obtained using a pulsed-tone stimulus and modified Hughson-Westlake procedure. Conventional PTA (at 0.25 kHz, 0.5 kHz, 1 kHz, 2 kHz, 4 kHz, 6 kHz, and 8 kHz, respectively) was performed at octave or semi-octave frequencies under air conduction and at octave frequencies from 0.25 to 4 kHz under bone conduction using a manual audiometer (GSI-Grason-stadler, USA) with TDH-50P supra-aural earphones (Telephonics, Farmingdale, USA). EHF audiometry (at 9 kHz, 10 kHz, 11.5 kHz, 12.5 kHz, 14 kHz, 16 kHz, 18 kHz and 20 kHz) was determined under air conduction using a calibrated audiometer (GSI-Grason-stadler, USA) with HDA-200 high frequency earphones (Sennheiser, Wedemark, Germany). The threshold was defined as the lowest decibel level at which the subject responded for at least 50% of the stimulus duration in ascending or descending presentations (ANSI, 2004) [19]. All audiometry equipment was calibrated according to ISO389-5 (International Organization for Standardization, 2006). A second measurement was performed to the participants 3 days after the initial test. Participants avoided the expose to loud noise (> 80 dB HL) 24 h prior to the test.

### Statistical analyses

Descriptive and quantitative analysis of difference associated with the independent variables were performed using SPSS 15 for Windows. Normality of the distribution was assessed using the Levine test. Comparisons of two independent samples (such as male versus female) were conducted either using two-tailed independent samples t-tests if the data were normally distributed revealed by the Levine test, or otherwise using the nonparametric rank-sum test (such as the hearing threshold). When the responsive rate to frequency didn’t exceed 20%, the hearing threshold value was not included. In all tests, *p* < 0.05 was considered to be significant.

## Results

### Study population

A total of 162 healthy participants were enrolled in this study, and grouped into five groups based on their ages: 21 ~ 30 years, 23 participants (46 ears) including 14 males and 9 females; 31 ~ 40 years, 25 participants (50 ears) with 16 males and 9 females; 41 ~ 50 years, 44 participants (88 ears) with 23 males and 21 females; 51 ~ 60 years, 39 participants (78 ears) with 19 males and 20 females; 61 ~ 70 years, 31 participants (62 ears) with 21 males and 10 females. There was no gender difference within each age group, nor individual difference between the right and the left ear on the pure-tone hearing thresholds at the frequencies from 0.25 to 20 kHz.

### Conventional pure tone audiometry

No reliable gender difference was seen in each group on hearing thresholds at the frequencies from 0.25 to 2 kHz. However, by merging different age groups, a significant difference (*p* < 0.05) on the mean thresholds was noticed between the 21 ~ 50 and 51 ~ 70 years old. The mean thresholds at 4 kHz was significantly different from each other when compared in pair (all *p* < 0.05), except for the comparison between the 21 ~ 30 and the 31 ~ 40 group. In addition, consistent threshold shifts at the frequencies of 6 and 8 kHz were seen among groups. The hearing thresholds at the frequencies of 6 and 8 kHz were significantly elevated in groups above 31 years old, and showed an age-dependency starting from 51 years old (*p* < 0.05). These findings suggested that hearing ability of healthy participants declined at the frequencies of 0.25 to 2 kHz from the age of 51, 4 kHz form the age fo 41, and 6 to 8 kHz from the age of 31. Table 1 depicted the hearing thresholds of subjects at various frequencies by age groups. The hearing ability at conventional high frequencies from 4 to 8 kHz declined from 51 years old, and the hearing thresholds ascended to 25 ~ 40 dB HL.

**Table 1:** The statistics of hearing thresholds at frequencies of subjects grouped by age

Frequency (kHz)	Hearing threshold (dB HL) M (QL, QU)				
	21–30 years	31–40 years	41–50 years	51–60 years	61–70 years
0.25–2	10.00 (6.50,10.00)	7.50 (5.00,11.25)	10.00 (8.75,15.00)	13.75 (10.00,22.50)^*△**▲**^	18.75 (11.25,32.50)^*△**▲**^
4	10.00 (6.50,10.00)	10.00 (5.00,20.00)	15.00 (10.00,25.00)^*^	25.00 (15.00,50.00)^*△**▲**^	40.00 (25.00,60.00)^*△**▲**■^
6	10.00 (6.50,10.00)	15.00 (7.50,35.00)^*^	17.50 (15.00,25.00)^*^	40.00 (15.00,60.00)^*△**▲**^	45.00 (35.00,55.00)^*△**▲**^
8	10.00 (6.50,10.00)	15.00 (5.00,30.00)^*^	20.00 (10.00,25.00)^*^	40.00 (15.00,60.00)^*△**▲**^	50.00 (35.00,60.00)^*△**▲**^
9	10.00 (10.00,15.00)	15.00 (10.00,47.50)^*^	27.50 (16.25,40.00)^*^	50.00 (28.75,75.00)^*△**▲**^	60.00 (55.00,70.00)^*△**▲**^
10	10.00 (10.00,15.00)	30.00 (12.50,45.00)^*^	32.50 (20.00,50.00)^*^	60.00 (28.75,75.00)^*△**▲**^	70.00 (60.00,80.00)^*△**▲**■^
11.5	10.00 (10.00,20.00)	40.00 (17.50,52.50)^*^	45.00 (30.00,63.75)^*^	62.50 (36.25,80.00)^*△**▲**^	75.00 (61.25,85.00)^*△**▲**■^
12.5	15.00 (10.00,20.00)	50.00 (25.00,65.00)^*^	55.00 (35.00,75.00)^*^	65.00 (51.25,83.75)^*△**▲**^	75.00 (70.00,85.00)^*△**▲**■^
14	20.00 (10.00,30.00)	60.00 (46.25,73.75)^*^	65.00 (50.00,80.00)^*^	70.00 (60.00,75.00)^*△**▲**^	75.00 (70.00,80.00)^*△**▲**^
16	25.00 (10.00,45.00)	50.00 (35.00,55.00)^*^	55.00 (50.00,60.00)^*△^	55.00 (55.00,60.00)^*△^	NA
18	20.00 (10.00,30.00)	NA	NA	NA	NA
20	10.00 (10.00,15.00)	NA	NA	NA	NA

*, significantly different (*p* < 0.05) from that of the 21–30 group

△, significantly different (*p* < 0.05) from that of the 31–40 group

▲, significantly different (*p* < 0.05) from that of the 41–50 group

■, significantly different (*p* < 0.05) from that of the 51–60 group

M (Q_L_, Q_U_), Median (lower quartile, upper quartile)

NA, not applicable since the response was equivalent or smaller than 20%

### Extended high frequency audiometry

**Fig. 1:**
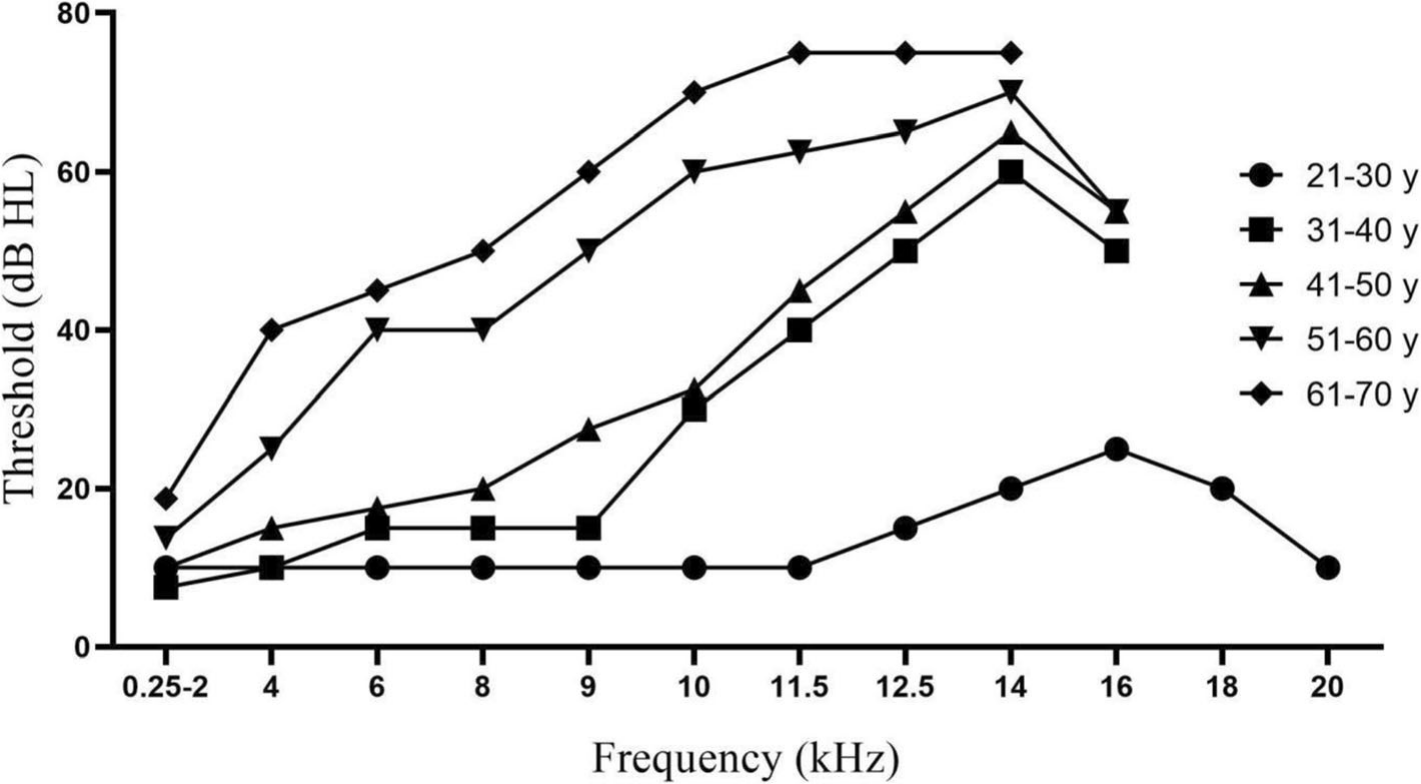
Overview of hearing thresholds at frequencies from 0.25 to 20 kHz of subjects grouped by age

The hearing thresholds in different age groups were compared and the results showed a significant difference at all frequencies from 9 to 16 kHz (*p* < 0.001) (Table 1). The hearing threshold at 9 kHz was significantly increased starting from the 31 ~ 40 group and worsened to 50 dB HL from 51 years old (*p* < 0.05), the same as the situation at the 6 and 8 kHz. At the frequencies of 10 kHz, 11.5 kHz, and 12.5 kHz, the average hearing thresholds between groups were significantly different from each other with the exception of the 31 ~ 40 group comparing with the 41 ~ 50 group. The hearing deterioration at the frequency of 14 kHz was quite similar to the changes at frequencies of 6, 8 and 9 kHz. At the frequency of 16 kHz, the average hearing threshold of 25 dB HL in the 21 ~ 30 group was significantly better than those in 31–60 age groups, and the threshold of 50 dB HL in the 31 ~ 40 group was better than those of 55 dB HL in the 41 ~ 60 age group (*p* < 0.05). These results showed that hearing at EHF degenerated in subjects starting from their 30s, and dominating in their 50s and older (Fig. 1). The hearing thresholds of EHF were less than 26 dB HL before 30 years old. The means of threshold values were up to 75 dB HL with aging. It is worth noting that less than 20% response was recorded at 16 kHz in the 61 ~ 70 group as well as at the 18 and 20 kHz in the groups older than 31 years old, suggesting that hearing ability declined at higher frequencies even in healthy population.

The sensitivity at EHF decreased as the frequency increased in each age group, and it decreased with aging for each frequency as well. Our results showed that all subjects aged below 30 years old were totally responsive at frequencies up to 16 kHz, while the responsiveness decreased to 52.2% at 20 kHz. More than 90% of the subjects aged 31 ~ 40 years old were totally responsive at frequencies up to 14 kHz, while it decreased below 50% at 18 kHz. More than 90% of the ears in the 41 ~ 50 group responded at frequencies up to 12.5 kHz, but comparatively similar percentage only occurred in the group older than 51 years old at frequencies up to 11.5 kHz. As shown in Table 2, these data demonstrated that the responsiveness of hearing in normal population decreased in a frequency and age dependent manner.

**Table 2:** NormalizedPercentage of responses at various frequencies

Frequency (kHz)	21–30 years (46 ears)	31–40 years (50 ears)	41–50 years (88 ears)	51–60 years (78 ears)	61–70 years (62 ears)
0.25–2	100.0	100.0	100.0	100.0	100.0
4	100.0	100.0	100.0	100.0	100.0
6	100.0	100.0	100.0	100.0	100.0
8	100.0	100.0	100.0	100.0	100.0
9	100.0	100.0	100.0	100.0	97.4
10	100.0	100.0	100.0	100.0	97.4
11.5	100.0	100.0	100.0	92.3	90.3
12.5	100.0	100.0	93.2	82.1	74.2
14	100.0	96.0	79.5	59.0	45.2
16	100.0	64.0	38.6	30.8	16.1
18	78.3	20.0	2.3	2.6	0.0
20	52.2	8.0	2.3	0.0	0.0

Moreover, no difference in the means of hearing thresholds between male and female subjects were seen nearly at all frequencies and in all age groups, except that gender differences (*p* < 0.05) could be found in the mean thresholds at frequencies of 4, 6, 8, and 9 kHz in the 41 ~ 50 group, suggesting a possibly greater hearing loss in males at their 40s.

## Discussion

Age related hearing loss is often gradual and progressed with subtle changes in an individual’s ability to discriminate high pitches. Meanwhile, some patients also complain about difficulty in understanding speech under noisy environments. Nevertheless, no apparent impact on verbal communication had been observed due to the hearing loss in EHF [3]. Therefore, delays in identification and subsequent help-seeking for hearing damage are quite common, which may prevent early diagnosis of hearing loss and are associated with physical and mental handicap [20]. Hearing screening has been suggested to be a potential useful tool to get around some of the deferments. The American Speech Language Hearing Association (2011) suggested that adults’ hearing should be screened in, at least, decade intervals before age 50 and in every 3 years thereafter, since individuals at their 50s might seem to have healthy hearing due to their normal conventional audiometric thresholds, yet the early onset of hearing impairment might happen.

We examined the hearing thresholds at frequencies from 0.25 to 20 kHz in the population claimed to have normal hearing abilities ranging from 20 to 70 years old in order to observe the potential age-related changes in their auditory system. As the hearing threshold at 4 kHz was obviously increased in adult older than 50 with clinically normal hearing sensitivity, subjects with hearing thresholds lower than 20 dB HL at up to 2 kHz were included in this study. Our data demonstrated similarities in average thresholds at frequencies from 0.25 to 2 kHz in subjects younger than 50 years old. However, the hearing thresholds increased predominantly starting from the age of 41 at 4 kHz, and from the age of 31 at 6 and 8 kHz. Therefore, we might conclude that the onset frequency of effects by aging were 4 kHz.

In this study, Fig. 1 demonstrated an overview of average hearing thresholds for each age group by frequencies. The average thresholds, which began to separate gradually from the adjacent groups at the frequencies below 14 kHz, were inversely proportional to the age of participants, possibly suggesting the aging of the auditory system. Moreover, our results showed that hearing ability at EHF declined beginning at 30 years of age, and became obvious at 50 years of age and later. These findings remarkably confirmed the notion that hearing thresholds at EHF could be more sensitive for early detection of the hearing damage than the conventional audiometric at lower frequencies [7, 21]. Meanwhile, it would be worth pointing out that the sensitivity of these EHF thresholds might decrease as the detecting frequency and participant’s age increasing, since we observed a decrease in the response to EHF above 14 kHz with an increase in both frequency and age. The percentage of response reached as high as 78.3% at 18 kHz and 52.2% at 20 kHz in their 20s. However, for groups with older participants, the percentage of response at 18 and 20 kHz did not exceed 20%, which is consistent with the previous investigations on subjects below 40 years old [22]. The overview of the responses at EHF of subjects ranging from 20 to 70 years old at a 10-year interval in the current study revealed the possibility that the clinical value of response to EHF might decrease as the frequency increase, and 14 kHz should be an optional choice.

Although studies suggested a potential gender difference in the hearing loss, with males earlier than females [23, 24], Wiley et al. reported hearing abilities among men and women were generally similar based on their observation of hearing thresholds at 0.5, 1, 2, and 8 kHz in a large population [25]. Consistently, here in our study, no significant gender difference of hearing thresholds within each age group was observed at most of the frequencies from 0.25 to 20 kHz, leaving only the 41 ~ 50 group at the frequencies of 4, 6, 8, and 9 kHz. However, it would still be plausible to claim that aging process of hearing system might be different in males and females, since other risk factors such as exposure to noise, smoking, and alcoholic abuse [19] that were excluded in the current study.

The current findings on thresholds at EHF, as well as previous studies describing their use, demonstrate the fact that EHF audiometry should be taken into consideration as a necessary and routine test to diagnose and monitor hearing damage. Early prediction and intervention in the potential population, such as medication dose change or proper use of hearing protection for the ototoxic effect and noise exposure, might ease or even cease the development of hearing loss [19, 26]. Furthermore, taking into consideration that hearing problems may exacerbate psycho-social declines, whereas age-related psycho-social issues may aggravate hearing impairment, it is important to further investigate hearing loss in aging adults and their effects. Therefore, our results on hearing thresholds at EHF in normal population might serve the need for future clinical diagnosis and laboratory research.

This study had limitations. The number of subjects from a single medical center was limited, and it could not fully reflect the epidemiological characteristics of age-related hearing changes. On the other hand, we detected the hearing thresholds at frequencies from 0.25 to 20 kHz in different age groups at the same period. In addition, no long-term follow-up was conducted for the same age group. Future research should involve multicentre cohort study over long term to determine the normal range of EHF deterioration. To establish the age-specific norm, more research is needed as well.

## Conclusion

This study revealed that hearing thresholds at EHF from 9 to 20 kHz were more sensitive than at frequencies below 8 kHz towards the prediction of hearing loss, and showed clear age-dependency. Both hearing thresholds at EHF and the upper-frequency limit deteriorated with aging and elevation of the frequency. The results of this study also demonstrated reproducibility in measuring hearing thresholds at EHF, indicating that the EHF audiometry might be a reliable tool in monitoring the early etiopathogenesis of auditory diseases.

## Data Availability

The raw datasets generated and analysed during the current study are available from the corresponding author on reasonable request.

## Abbreviations

EHF: Extended high frequency
PTA: Pure tone average
NIHL: Noise-induced hearing loss
